# Epidemic Cholera in the Peninsula of Yucatan

**Published:** 1833

**Authors:** 


E Sylvis nuncius.

---

*Cincinnati, January* 1, 1834.

---

### I. Epidemic Cholera in the Peninsula of Yucatan.

Dr. Perrine's interesting letter on the Cholera as it appeared in Campeche, and other parts of Yucatan, which makes the initial article of this number, is, we believe, the first account of the Epidemic in Spanish America. We feel the more pleasure in presenting it to our readers, as its ingenious author commenced his professional career in the valley of the Mississippi; where he brought himself into notice by the boldness and success of his practice in congestive intermittents, which he treated with doses of the sulphate of quinine, far greater than had ever before been administered. It should be gratifying to the physicians of the United States, that they had, during the prevalence of the Cholera in a distant part of the New World, a representative, so well qualified to establish the superiority of their practice, over that of the French and Spanish physicians, by whom he was surrounded. As an evidence of the estimate in which his services were held, by the people among whom they were rendered, we may mention, that the public authorities of Campeche, expressed their feelings, not only by a letter of thanks, but a request to the congress to grant him the title of Mexican citizen.

